# Micronutrient Inadequacy in Short Sleep: Analysis of the NHANES 2005–2016

**DOI:** 10.3390/nu11102335

**Published:** 2019-10-01

**Authors:** Chioma J. Ikonte, Jonathan G. Mun, Carroll A. Reider, Ryan W. Grant, Susan Hazels Mitmesser

**Affiliations:** Science & Technology, Pharmavite LLC, West Hills, CA 91304, USA; cikonte@pharmavite.net (C.J.I.); creider@pharmavite.net (C.A.R.); rgrant@pharmavite.net (R.W.G.); smitmesser@pharmavite.net (S.H.M.)

**Keywords:** short sleep, sleep duration, sleep quality, micronutrient intake, micronutrient, inadequate intake, NHANES, adults, nutrient, EAR

## Abstract

One third of U.S. adults report short sleep (<7 h), which has been linked to negative health outcomes. Inadequate intake of micronutrients across the U.S. adult population has been reported, and a relationship between sleep conditions and micronutrient intake is emerging. This cross-sectional analysis of the National Health and Nutrition Examination Survey (NHANES 2005–2016) (*n* = 26,211) showed that participants with short sleep duration had a lower usual intake (Food + Supplements) of calcium, magnesium, and vitamin D in all adults aged 19+ years, and vitamin K in adults aged 19–50 years, even after adjusting for covariates. In addition, participants reporting short sleep had a higher percentage of individuals with intake lower than the estimated average requirement (EAR) across multiple nutrients. Age and gender differences were observed in the prevalence of inadequate intake across multiple nutrients. Adults aged 51–99 years with short sleep duration had inadequate intake with respect to more nutrients. In females there was an association between short sleep and a higher prevalence of inadequate intake (Food + Spp) for calcium, magnesium, and vitamins A, C, D, E, and K (above adequate intake). Conversely, males reporting short sleep only had an inadequate intake of vitamin D. Overall, we demonstrate that short sleep is associated with increased nutrient inadequacy, emphasizing the possible need for dietary supplementation.

## 1. Introduction

By 2030, the cost of insufficient sleep has been projected to reach up to US$467.7 billion, primarily due to lower productivity and high mortality risk linked to insufficient sleep [1]. As part of Healthy People 2020, the Centers for Disease Control and Prevention (CDC) has partnered with the American Academy of Sleep Medicine (AASM) to improve sleep health and increase the number of Americans getting sufficient sleep. While sleep deprivation has been linked to irritability, cognitive impairment, memory lapses or loss, and an impaired immune system, adequate sleep duration has a restorative effect on the body, particularly the immune system, endocrine system, and nervous system. Thus, the leading sleep organizations encourage adequate sleep as an important component of selfcare and healthcare. The National Sleep Foundation defines adequate sleep as 7–9 h for adults aged 26–64 years and 7–8 h for older adults aged ≥65 years, and insufficient sleep as <6 h for adults and 5–6 h for older adults [2]. In addition, a joint consensus statement of the American Academy of Sleep Medicine and Sleep Research Society recommends seven or more hours of sleep per night on a regular basis to promote optimal health among adults aged 18–60 years [3]. Unfortunately, most Americans fall short of this recommendation. Self-reported sleeping hours among U.S. adults based on the National Health and Nutrition Examination Survey (NHANES) 2007–2010 show that an estimated 37.3% of U.S. adults are receiving 6 h or less of sleep per night, while only 60.4% report sleeping 7–9 h [4].

Reduced sleep duration has been associated with biomarkers of disease. For instance, self-reported short sleep has been shown to alter cardiometabolic disease risk factors [5] including higher systolic and diastolic blood pressure [6], inflammation [7], impaired glucose tolerance [8], higher serum TG, lower serum HDL cholesterol [9], and hypertension, particularly in premenopausal women [10]. Additionally, a number of cross-sectional studies have reported an association between short sleep duration and higher prevalence of weight gain or obesity in children and adults [11,12,13]. Given the association between short sleep and risk factors of cardiometabolic disease, it is not surprising that self-reported short sleep duration is also associated with all-cause mortality [14], irrespective of weight status. It is well known that diet also impacts cardiometabolic risk factors, and there is growing interest on the impact of sleep on diet and vice versa, and their association with chronic disease risk.

Many researchers have investigated causes and risk factors for short sleep, including poor sleep hygiene, ethnic disparities, and environmental/lifestyle factors, namely nutrition. Epidemiological studies have begun to link short sleep duration and other sleep conditions to nutrient intake. Using the National Health and Nutrition Examination Survey (NHANES 2007–2008) database, Grander et al. [15] demonstrated an association of sleep symptoms with intake of several dietary nutrients. In addition, they also reported that individuals with very short sleep duration (<5 h) demonstrated the lowest intake of all macronutrients (protein, carbohydrates, sugars, dietary fiber, and fat) and women were more likely to have very short sleep (<5 h) or long sleep (>9 h) duration compared to men [15]. While these observations provide insights on the association of nutrient intake with sleep conditions across the U.S. population, they do not address the association of sleep conditions with inadequate micronutrient intake. Many of the nutrients associated with sleep conditions have also been identified as shortfall nutrients due to the high number of individuals not meeting adequate intake. Blumberg et al. [16] found a high prevalence of inadequacy for vitamin D (96%), vitamin E (87%), magnesium (55%), calcium (41%), vitamin A (48%), and vitamin C (46%) [16]. The 2015–2020 Dietary Guidelines for Americans (DGAs) identified potassium, dietary fiber, choline, magnesium, calcium, and vitamins A, D, E, and C as nutrients consumed below recommended levels and reported vitamin D, calcium, potassium, and fiber as nutrients of public health concern [17]. As a result, the objective of this study was to investigate the relationship between short sleep and micronutrients intake and inadequacy in U.S. adults aged 19+ years using the most up-to-date dataset of the nationally representative database, the NHANES 2005–2016.

## 2. Materials and Methods

### 2.1. Database and Sample

The study dataset includes participants from the last six cycles of the National Health and Nutrition Examination Survey (NHANES) 2005–2016. Detailed descriptions of the subject recruitment, survey design, and data collection procedures for NHANES are available online (http://www.cdc.gov/nchs/nhanes/about nhanes.htm). NHANES data are currently collected continuously using a stratified multistage cluster sampling probability design and data are released every two years. Participants are interviewed in their homes for demographic (age, gender, and race/ethnicity), socioeconomic (family income, and education level), and general health information (current smoking and alcohol intake), and subsequently receive a comprehensive health examination conducted in a mobile examination center (source of body mass index data) [4].The NHANES study protocol was approved by the Research Ethics Review Board of the National Center for Health Statistics and all participants provided written informed consent. After excluding those pregnant/lactating and those with unreliable dietary records, the overall sample size was 26,211. Poverty income ratio (PIR) was determined as ratio of family income to the poverty level threshold (maximum determined as 5.0); out of which three levels of PIR were derived: ≤1.35, >1.35 to <1.85, and ≥1.85. Education level was also classified as three levels: <high school graduate, high school graduate, and more than high school graduate. Current smoking was defined as “yes” or “no” and alcohol intake was defined as average drinks/day.

### 2.2. Sleep Variable—Short Sleep

The response to the NHANES question: “How much sleep do you usually get at night on weekdays or workdays?” was available for all six cycles of NHANES 2005–2016 and used to assess sleep hours, with short sleep duration defined as <7 h per night.

### 2.3. Usual Nutrient Intakes

Dietary intake was determined using two reliable 24-h dietary recall interviews using the United States Department of Agriculture (USDA) automated multiple-pass method (AMPM) [4]; the first recall was conducted in person, while the second recall was conducted via a phone call. The nutrient content of foods consumed by NHANES 2005–2016 participants was determined by using relevant Food and Nutrient Database for Dietary Studies (FNDDS) for each NHANES release [18,19]. A dietary supplement questionnaire assessing the usage of vitamins, minerals, botanicals, and other dietary supplements was administered as part of the NHANES household interview, and the consumption frequency, duration, and dosage were recorded for each supplement used over the prior 30 days [20]. The complete product information including labeled dosage or serving size, ingredients, and the amounts of ingredients per serving was recorded when possible. The average daily intake of nutrients from dietary supplements was calculated using the supplement consumption frequency and dosage.

Individual usual nutrient intakes (long-term intakes) from “Food Only” and from “Food Plus Supplement” (Food + Spp) were estimated using two days of dietary intake in NHANES with the National Cancer Institute method [21] and with day of recall, weekday/weekend intake flag, and dietary supplement use (yes/no) flag as covariates. Given dietary supplement data were not available for 2015–2016, individual usual intakes including dietary supplements only used NHANES data for 2005–2014. The percentage of the population below the estimated average requirement (EAR) was determined using the cut-point method (except for iron where the probability method was used) for 17 nutrients (calcium, copper, iron, magnesium, phosphorus, selenium, zinc, vitamin A, thiamin, riboflavin, niacin, folate, vitamin B6, vitamin B12, vitamin C, vitamin D, and vitamin E). Additionally, the percentage of population receiving above the adequate intake (AI) for three nutrients (potassium, vitamin K and choline) was assessed.

### 2.4. Statistics

Analyses used the SAS 9.4 (SAS Institute, Inc.; Cary, NC, USA) survey procedures to account for the complex survey design of NHANES and all analyses used appropriate sample weights [22]. Mean ± standard error of the mean (SEM) for various demographic variables were determined for the overall population for genders combined (all adults) and males/females separately. Regression analyses were conducted to assess differences in individual usual intakes (means ± SE) from Food Only and from Food + Spp among those with and without short sleep duration. Additional regression analyses were conducted to assess differences in the percentage of the population below the EAR or above the AI among those with and without short sleep. Subsequently, further regression analyses were conducted adjusting individual usual intake (IUI) from Food + Spp using the following covariates: age, gender, race/ethnicity, PIR level, education level, BMI, current smoking status, physical activity level (classified as sedentary, moderate, and vigorous based on responses to physical activity questions), IUI of alcohol, and IUI of energy. Given the large sample size, a *p*-value of <0.01 was used to assess statistical differences.

## 3. Results

### 3.1. Demographics of the Participants

The demographics of the study participants is summarized in Table 1. All samples are weighted. The average age of the participants was 47 years, with slightly more females than males (51% vs. 49%). About two-thirds of the participants were Non-Hispanic White, while Non-Hispanic Blacks and Mexican Americans represented about 11% and 9% of the participants, respectively. The reported average sleep time was 7 h and about 20% adults were current smokers. The study participants reported a high prevalence of short sleep. About one third reported short sleep duration, with slightly more male than female participants reporting short sleep (34.42% vs. 31.08%). The participant demographic distribution with and without short sleep duration was reported in detail by Ikonte et al. (2019, manuscript under review). Specifically, significant differences between sleep variables and age, gender, race/ethnicity, PIR level, education level, BMI, and current smoking status were observed (see Table 1).

### 3.2. Micronutrient Usual Intake (UI) (Food Only and Food + Spp) and Inadequacy (% Below EAR) With and Without Short Sleep in All Adults Aged 19+ years

From Food Only, the UI of calcium, copper, folate, magnesium, potassium, thiamin, and vitamins A, C, D, E, and K was significantly lower in all adults with short sleep duration. Usual intake from Food + Spp of calcium, copper, folate, iron, magnesium, niacin, potassium, zinc, and vitamins A, C, D was significantly lower in all adults reporting short sleep duration. After adjusting Food + Spp UI for covariates in all adults, calcium, magnesium, and vitamin D remained statistically significant (Table 2).

In all adults, short sleep duration was significantly associated with a higher percentage of the population with intake from Food Only below the EAR for copper, folate, iron, magnesium, riboflavin, zinc, and vitamins A, C, E, and K (above the AI). Similarly, short sleep was significantly associated with higher percentage of the population with intake from Food + Spp below the EAR for calcium, copper, folate, iron, magnesium, riboflavin, thiamin, zinc, and vitamins A, C, D, E and K (above AI). Regardless of the source of intake (Food Only or Food + Spp), we found a significant association between short sleep and the percentage of the population with intake below the EAR for copper, folate, iron, magnesium, riboflavin, zinc, and vitamins A, C, and K (above AI) in all adults (Table 2).

Further analysis of the data by age group showed a significant association of short sleep duration with UI (Food Only) for calcium, copper, magnesium, and vitamins A, C, E, and K in adults aged 19–50 years (Table S1), and with UI for copper, folate, magnesium, potassium, and vitamins A, C, D, E, and K in adults aged 51–99 years (Table S2). Additionally, short sleep was significantly associated with UI (Food+Spp) of copper, folate, magnesium, vitamin D, potassium, and vitamin K in adults aged 19–50 years, and with UI of calcium, copper, folate, iron, magnesium, niacin, phosphorus, potassium, selenium, and vitamin A in adults aged 51–99 years (Tables S1 and S2). After adjusting Food + Spp UI for covariates, magnesium and vitamins D and K remained significant in adults aged 19–50 years (Table S1), while only calcium remained significant in adults aged 51–99 years (Table S2). In addition, we found that short sleep duration was significantly associated with a higher percentage of the population with inadequate intake (Food Only) of calcium, copper, folate, magnesium, thiamin, and vitamins A, C, E, and K (above AI) in adults aged 19–50 years (Table S1). For Food + Spp, intake below the EAR of calcium, copper, folate, magnesium, thiamin, and vitamins A, C, D, and K (above AI) in adults aged 19–50 years was associated with short sleep duration (Table S1). Among adults 51–99 years, short sleep duration was significantly associated with a higher percentage of the population with intake below the EAR for copper, magnesium, riboflavin, and vitamins A, C, E and K (above AI) from Food Only and calcium, copper, folate, magnesium, riboflavin thiamin, zinc, and vitamins A, B6, C, D, E, and K (above AI) from Food + Spp (Table S2). Across multiple nutrients showing significant association with the presence of short sleep, we report a higher percentage of the population with intake below the EAR.

**Figure 1:**
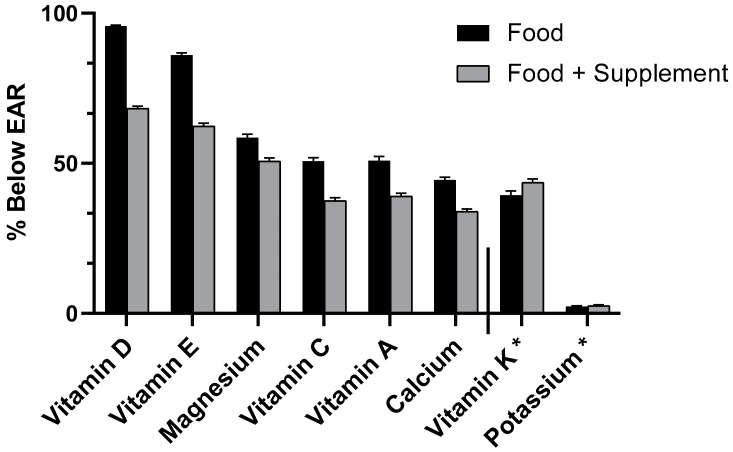
Prevalence of inadequacy (% of population below EAR) from Food Only and Food + Spp, all adults aged 19+ years with short sleep duration (<7 h). *% above adequate intake (AI).

For a number of shortfall nutrients showing associations with short sleep, we found a lower percentage of the population receiving below the EAR when nutrient intake included supplementation in addition to foods, although no statistical comparison was made. The percentages of all adults with short sleep duration receiving below the EAR (Food Only and Food + Spp) are as follows: vitamin D (96% and 68%), vitamin E (86% and 62%), magnesium (59% and 51%), vitamin C (51% and 38%), vitamin A (51% and 39%), calcium (44% and 34%), vitamin K (above AI, 39% and 44%), and potassium (above AI, 2% and 3%), respectively (Figure 1).

### 3.3. Micronutrient Usual Intake (UI) (Food Only and Food + Spp) and Inadequacy (% Below EAR) With and Without Short Sleep in Females Aged 19+ years

Among all females with short sleep, we found a lower UI (Food Only) of calcium, copper, folate, iron, magnesium, phosphorus, potassium, riboflavin, selenium, thiamin, choline, zinc, and vitamins A, B6, C, D, E, and K. Among these females with short sleep duration, we found lower UI (Food+Spp), of calcium, copper, folate, iron, niacin, phosphorus, potassium, choline, zinc, and vitamins A and D. Regardless of the source of nutrient intake (Food Only or Food + Spp), there was significantly lower intake (UI) of calcium, copper, folate, phosphorus, potassium, choline, iron, zinc, and vitamins A and D in females with short sleep duration(Table 3). After adjusting for covariates Food + Spp UI, calcium and phosphorus remained significant among females aged 19–99 years with short sleep duration.

We show that females with short sleep had a higher prevalence of inadequate intake from Food Only of calcium, copper, folate, iron, magnesium, riboflavin, thiamin, zinc, and vitamins A, C, E and K (above the AI). Similarly, from Food + Spp, females reporting short sleep had a higher population with intakes below the EAR of calcium, copper, folate, iron, magnesium, riboflavin, thiamin, vitamins A, B6, C, D, E, and K (above AI), and zinc. Regardless of nutrient source (Food Only or Food + Spp), short sleep was associated with a higher prevalence of intake below the EAR of calcium, copper, folate, iron, magnesium, riboflavin, and vitamins A, C, E, and K (above AI) among all females (Table 3).

Further analysis of the data by age group showed that females 19–50 years with short sleep had lower UI of calcium, copper, folate, iron, magnesium, niacin, phosphorus, potassium, riboflavin, selenium, thiamin, choline, vitamins A, B6, C, D, E, and K (above AI), and zinc from Food Only and lower UI of copper, calcium, phosphorus, potassium, choline, and vitamin K from Food + Spp (Table S3).

Females aged 51–99 years reporting short sleep had lower UI of calcium, copper, folate, iron, magnesium, phosphorus, potassium, riboflavin, selenium, thiamin, choline, vitamins A, B6, C, D, E, and K (above AI), and zinc from Food Only, and lower UI of calcium, copper, folate, magnesium, niacin, phosphorus, potassium, vitamin A, and zinc from Food + Spp (Table S4). Regardless of age group and source of nutrient intake (Food Only or Food + Spp), females with short sleep had reduced intake of calcium, copper, folate, magnesium, potassium, phosphorus, vitamin A, and zinc (Table S4). Among females aged 51–99 years, only calcium, phosphorus, and potassium remained statistically significant after adjusting for covariates in Food + Spp UI (Table S4), while no nutrients remained significant in females aged 19–50 years (Table S3).

Among females aged 19–50 years with short sleep duration, we found a higher percentage of the population with intake below the EAR (Food Only and Food + Spp) of calcium, copper, folate, iron, magnesium, riboflavin, thiamin, zinc, and vitamins A, C, and K (above AI), and additionally niacin and vitamin E from Food Only (Table S3).

Similarly, in females aged 51–99 years with short sleep there was an association with the percentage of the population with intake below the EAR (Food Only and Food + Spp) for calcium, copper, folate, magnesium, riboflavin, thiamin, zinc, and vitamins A, B6, C, and K (above AI), and additionally vitamin E from Food Only (Table S4).

### 3.4. Micronutrient Usual Intake (UI) (Food Only and Food + Spp) and Inadequacy (% Below EAR) With and Without Short Sleep Duration in Males Aged 19+ years

Our data show that males aged 19–99 years with short sleep had lower UI of copper, folate, niacin, and vitamins D and K from Food + Spp, and lower UI of magnesium and vitamins D and K from Food Only. After adjusting for covariates, for Food + Spp UI no nutrients remained significant (Table 4).

Further analysis by age group showed males aged 19–50 years with short sleep had a lower UI from Food + Spp of vitamin B12 and thiamin. No significant association was found between the UI from Food Only of any nutrient and short sleep among these male participants. After adjusting for covariates for Food + Spp UI, only thiamin remained statistically significant (Table S5) among males aged 19–50 years. Males aged 51–99 years reporting short sleep had lower UI from Food + Spp of copper and niacin. We found no significant association between the UI from Food Only of any nutrient and short sleep in males aged 51–99 years (Table S6).

In all males aged 19–99 years reporting short sleep duration, we observed a higher percentage of the population with inadequate intake from Food Only of magnesium, vitamins A and K (above AI) (Table 4), and for Food + Spp, we found a higher percentage of the population with inadequate intake of magnesium and vitamins C, D, E and K (above AI) (Table 4).

Further age group analysis showed that among male participants aged 19–50 years with short sleep there was a higher percentage of the population with intake below the EAR (Food Only and Food + Spp) for magnesium. Males aged 51–99 years reporting short sleep showed a higher percentage of the population receiving below the EAR (Food+Spp) for magnesium and vitamins C and D.

## 4. Discussion

Our data demonstrate an association between the presence of short sleep and the percentage of the population below the EAR for multiple nutrients. For both groups (Food Only and Food + Spp), a greater percentage of all adults reporting short sleep had inadequate intake of copper, folate, iron, magnesium, riboflavin, zinc, and vitamins A, C and K. Almost one third of the U.S. population is at risk of a vitamin deficiency, while individuals who meet adequate micronutrient intake have a lower risk of deficiency [23]. Accordingly, the U.S. Dietary Guidelines has identified suboptimal intake of several nutrients, and has further classified calcium, vitamin D, potassium, and fiber as nutrients of public health concern because of their link to negative health outcomes [17]. An analysis by Fulgoni et al. shows shortfalls for calcium (48.9%), magnesium (54.5%), vitamin A (45.1%), vitamin C (37.0%), vitamin D (93.3%), vitamin E (90.7%), and vitamin K (31.1%) using the NHANES 2003–2006 [24]. Our data, represented as percentages below EAR, show that groups reporting short sleep had inadequate intake of several nutrients including nutrients classified as “under-consumed”. In addition, we highlight gender and age differences in the percentage of the population below EAR reporting short sleep. Across age groups (19–50 years and 51–99 years) we found differences in the association between short sleep and the prevalence of inadequate intakes (% below EAR). We show that the number of nutrients with inadequate intake among participants with short sleep was greater in older adults aged 51–99 years (Tables S1 and S2), emphasizing a need for supplementation with age. We also note gender differences in the number of nutrients with inadequate intake among individuals reporting short sleep. For females reporting short sleep, an association with the percentage of the population with intake below EAR for calcium, magnesium, and vitamins A, C, E, and K (above AI) (Food Only and Food + Spp), and vitamin D (Food + Spp) was found. Males reporting short sleep had inadequate intake of only vitamin D (Food + Spp).

Previous research exploring associations between sleep and diet has focused only on nutrient intake, but not on the relationship between nutrient inadequacy and sleep. In the analysis of the NHANES 2007-2008 dataset, Grandner et al. [15] reported an association between a decreased intake of several nutrients and multiple sleep symptoms. Specifically, they reported lower intake of selenium with increased difficulty falling asleep and lower intake of vitamin C with non-restorative sleep. While a higher calcium intake was associated with decreased difficulty falling asleep and decreased non-restorative sleep, higher vitamin D intake was associated with less difficulty maintaining sleep and higher potassium intake was associated with less daytime sleepiness [15]. Consistent with previous reports, we demonstrate significant associations between usual intake of individual shortfall nutrients and short sleep. Additionally, we report significant associations between the percentage of the population below the EAR for these shortfall nutrients and short sleep.

### 4.1. Vitamin D

Our results show that participants reporting short sleep had lower usual intake (Food + Spp) of vitamin D after adjusting for covariates. In addition, we report a significant association between the presence of short sleep and the percentage of population below EAR for vitamin D, with 68% of all adults receiving below the EAR for vitamin D reporting the presence of short sleep (Table 2).

The association between the percentage of the population below EAR for vitamin D and short sleep was significant in both age ranges (19–50 years and 51–99 years). This finding is supported by Massa et al., who reported a dose-dependent significant trend (P_trends_ < 0.001) between lower levels of 25-hydroxyvitamin D and higher odds of short sleep, poorer sleep efficiency, and increased sleep fragmentation in older men (>68 years) [25]. They reported that men with the lowest serum concentrations of 25 (OH)D (<20 ng/mL and 20–30 ng/mL) had a 2-fold increase in the odds of short sleep duration compared to men with the highest serum 25 (OH)D (30–40 ng/mL and ≥40 ng/mL) levels. Similarly, a cross-sectional study by Piovezan et al. reported that in older adults (50+ years), obstructive sleep apnea and objective short sleep duration are independently associated with risk of serum vitamin D deficiency [26]. The NHANES 2007–2008 study by Grandner et al. [15] showed an association between low vitamin D intake (odds ratio = 0.84) and difficulty maintaining sleep.

In the Multi-Ethnic Study of Atherosclerosis (MESA), shorter sleep duration (373.3 ± 80.7 min) measured by polysomnography was significantly associated with 25 (OH)D serum levels lower than 20 ng/mL, while a lower proportion of rapid eye movement (REM) sleep was weakly correlated with 25 (OH)D values between 20 and 29 ng/mL [27]. A recent meta-analysis indicated that vitamin D deficiency is associated with higher risk of sleep disorders [28]. Participants with vitamin D deficiency had significantly increased risk of sleep disorders (odds ratio = 1.50) compared with high vitamin D status. In a sub-group analysis, a significant association was found between vitamin D deficiency and short sleep duration [28]. Involvement of vitamin D in sleep regulation has been shown [29], and vitamin D receptors have been identified in regions of the brain that regulate sleep, including the hypothalamus, prefrontal cortex, mid-brain central gray, substantia nigra, and raphe nuclei [30,31]. Calcitriol administration in cell cultures affected the expression of two genes involved in circadian rhythm (BMAL1 and PER2) over a 60-h period, suggesting the possible role of vitamin D in regulating genes that are involved in circadian rhythm [32].

Additional evidence suggests that vitamin D may be involved in decreased release of inflammatory substances including prostaglandin D2 and tumor necrosis factor alpha (TNF-a) which play roles in sleep regulation [33]. With the potential role of vitamin D in sleep regulation, it not surprising that our data show a significant association between short sleep and inadequate intake of vitamin D.

### 4.2. Magnesium

We show a significant association between the usual intake (Food+Spp) of magnesium in all adults with short sleep even after adjusting for covariates. In addition, we report the association between the presence of short sleep and the percentage of all adults with intake below the EAR for magnesium (59% and 51% for Food Only and Food + Spp; respectively; Table 2). The association with inadequate intake (Food + Spp) of magnesium with short sleep persisted across all age groups (19–50 years and 51–99 years) of adults, and separately for males and females (Tables S1–S6). In all adults with short sleep, supplementation decreased the prevalence of inadequate intake (Table 2). In older adults (aged 51–99 years), the prevalence of inadequate magnesium intake decreased from 60% to 49% from supplementation (Table S2).

Previous research has reported links between magnesium and sleep. Magnesium deficiency in rodent models showed alterations in sleep organization and wakefulness that were reversed upon reintroduction of dietary magnesium [34]. An analysis of the 2007–2008 NHANES found that lower magnesium intake was associated with very short (<5 h) sleep [15]. A cohort study of 1056 participants recruited into the Jiangsu Nutrition Study (JIN) from 2002 to 2007 found that dietary magnesium consumption was inversely associated with falling asleep during the day in women but not in men [35], which parallels our findings in night time sleep duration. Abbasi et al. demonstrated in a double-blind placebo-controlled trial with elderly subjects who received 500 mg magnesium or placebo daily for 8 weeks that magnesium supplementation increased sleep time, sleep efficiency, and serum melatonin concentration, and decreased Insomnia Severity Index score, sleep onset latency, and serum cortisol concentration compared to placebo [36].

Although the specific mechanisms by which magnesium supports sleep is not fully understood, studies have shown that magnesium plays essential roles in the sleep–wake cycles in the body. Daily magnesium fluxes are thought to regulate cellular timekeeping and energy balance [37]. Intracellular magnesium concentration acts as a cell-autonomous timekeeping component to determine key clock properties in human cell lines and unicellular alga [38]. In addition, magnesium has an essential role in ion transport and electrical conductivity, which facilitate N-methyl-D-aspartic acid (NMDA) receptor function, an important sleep regulator [36]. Magnesium may also play a role in melatonin synthesis as a cofactor for serotonin N-acetyltransferase (arylalkylamone-N-acetyltransferase; AANAT), which facilitates the conversion of serotonin to N-acetylserotonin, the rate limiting step in melatonin synthesis [39]. Overall, these findings highlight the significance of magnesium in sleep health and the need for adequate intake of this nutrient.

### 4.3. Vitamins C and E

We show a greater percentage of all adults reporting short sleep had inadequate intake (Food Only and Food + Spp) for vitamin C, with 51% receiving below the EAR from Food Only and 38% receiving below the EAR from Food + Spp (Table 2). The association with inadequate intake (Food Only and Food + Spp) of vitamin C with short sleep persisted across all age groups (19–50 years and 51–99 years) of adults and females alone (Tables S1–S4), and in males aged 51–99 years (Food+Spp) (Table S6). In all adults, supplementation decreased the prevalence of inadequate intake for vitamin C (Table 2). In adults (51–99 years), the prevalence of inadequate vitamin C intake decreased from 47% to 30% from supplementation (Table S2). These results are supported by Grandner et al. who found an association between vitamin C and other nutrients with sleep symptoms [40], particularly short sleep duration [15]. The majority of dietary vitamin C is derived from fruits and vegetables, and their intake is positively associated with plasma antioxidants [41]. Additionally, Stamatakis et al. associated low fruit and vegetable intake with short sleep [42]. Further work using the UK-based National Diet and Nutrition Survey found an association between lower plasma vitamin C and sleep duration [43]. We have also found that smoking status was associated with increased short sleep (Ikonte, TBD/J Sleep Res. 2019, manuscript under review); smoking creates oxidative stress that is known to deplete antioxidants, vitamin C, and vitamin E. Interestingly, we report an association between the percentage of the population below the EAR (Food+Spp) for vitamin E and the presence of short sleep in all adults, and in males and females separately. Among all adults reporting short sleep, 86% of the population received below the EAR from Food Only for vitamin E and 62% from Food + Spp. As a lipid-soluble antioxidant, vitamin E may have neuroprotective roles. A rodent model demonstrated a neuroprotective role of vitamin E on chronic sleep deprivation-induced memory impairment [44]. Together, these data demonstrate that essential antioxidant intake (vitamin C and vitamin E) is reduced and inadequacy is increased with short sleep. Clinical studies are needed to determine if raising antioxidant status improves sleep duration or other sleep symptoms.

### 4.4. Vitamin A

We found a significant association between the presence of short sleep and the percentage of all adults below the EAR (Food Only and Food + Spp) for vitamin A, with 51% below the EAR from Food Only and 39% below the EAR from Food + Spp (Table 2). The association between inadequate intake (Food Only and Food + Spp) of vitamin A and short sleep persisted across all age groups (19–50 years and 51–99 years) of adults, and females (Tables S1–S4). In all adults, supplementation decreased the prevalence of inadequate intake for vitamin A (Table 2). Among adults aged 19–50 years and 51–99 years with short sleep, the prevalence of inadequate vitamin A intake decreased from 55% to 44% and from 45% to 30%, respectively, with supplementation (Table S2). Previous studies have demonstrated that short sleep is associated with lower carotenoid intake [43], and that low serum carotenoid concentrations are associated with higher odds of short sleep [45]. In the eye, vitamin A and pro-vitamin A carotenoids contribute to the sensing of light, which is important for entrainment of circadian rhythms. The mammalian retina contains three classes of photoreceptors that convert light information into electrical signals—rods, cones, and intrinsically photosensitive retinal ganglion cells. Photoreceptors contain light-sensing opsin proteins. Within retinal ganglion cells, melanopsin plays a key role in circadian photoreception [46]. Opsin proteins rely on the cyclical regeneration of a light-sensing chromophore, 11-cis-retinal. The generation of 11-cis-retinal by the retinoid cycle is dependent on dietary carotenoids and retinoids (vitamin A). Additionally, the nuclear receptor responsible for retinoic acid signaling (retinoic acid receptor alpha) is expressed in the human suprachiasmatic nucleus and shows circadian rhythmicity of mRNA expression in rats [47]. The vasopressin neurons that express retinoic acid receptor-alpha in the hypothalamus have been hypothesized to stabilize the circadian clock, possibly through a retinoic acid-dependent mechanism which inhibits the expression of circadian clock master genes [47]. Together, these results indicate that U.S. adults experiencing short sleep have higher prevalence of inadequate vitamin A intake, and that vitamin A plays a role in circadian physiology. Because dietary recommendations are based on the prevention of deficiency, future clinical studies are needed to determine if vitamin A status can be optimized for sleep health benefits.

### 4.5. Calcium

We found a significant association between the usual intake (Food+Spp) of calcium in all adults with short sleep, even after adjusting for covariates. In addition, we report the association between the presence of short sleep and the percentage of all adults and separate females with intake below the EAR for calcium. Thirty-four (34%) of all adults reporting short sleep were receiving below the EAR for calcium (Food+Spp). Among females reporting short sleep, 62% (Food Only) and 46% (Food+Spp) received below the EAR for calcium (Table 2 and Table 3).

The association between inadequate intake (Food+Spp) of calcium and short sleep persisted across all age groups (19–50 years and 51–99 years) of adults and females alone, but not males (Tables S1–S6). In all adults with short sleep, supplementation decreased the prevalence of inadequate intake of calcium (Table 2). In older females (aged 51–99 years), the prevalence of inadequate calcium intake decreased from 79% (Food Only) to 53% (Food+Spp) (Table S2). Calcium is the most abundant mineral in the body, yet the 2015–2020 Dietary Guidelines for Americans (2015 DGA) consider it as an under-consumed nutrient of public health concern, especially in the female population. While the body of research on calcium focuses mainly on bone health, there is little research looking at the relationship between calcium intake and sleep. Grandner et al. found calcium to be independently associated with difficulty falling asleep (OR = 0.83) and non-restorative sleep (OR = 0.81) [40], proposing that fewer sleep difficulties associated with higher calcium intake may contribute to lowering blood pressure [40,48]. Additionally, other research showed calcium along with magnesium and potassium act as neuromodulators in the sleep/wake cycle and may play an important role in melatonin production through the activation of tryptophan hydroxylase [49,50].

Emerging research has suggested that poor sleep may be an unrecognized risk factor for bone loss. Cunningham et al. reviewed NHANES 2005-2006 and 2007-2008 and found shorter sleep duration (<6 h) was significantly associated with osteoporosis after adjusting for potential covariates (odds ratio (OR) = 1.59). After stratifying by age group, this association remained significant (OR = 1.80) only in the older age group (>65) [51]. Chronic sleep deprivation impacts bone mass and bone metabolism (decreases bone mineral density, bone volume over total volume, trabecular bone thickness, and trabecular bone number; increases bone surface area over bone volume and trabecular bone separations [52]. Consistent with the above, our findings show significant association between short sleep and nutrients associated with bone health, including calcium, magnesium, vitamin D, and vitamin K. In all adults 19+ years, even after adjusting for covariates, the usual intake (Food+Spp) of calcium, magnesium, and vitamin D (and in addition vitamin K for adults aged 19–50 years) remained significantly associated with short sleep duration. Alarmingly, 79% of females aged>51 years who experienced short sleep had an inadequate intake of calcium (Food Only). Additionally, individuals in this group had inadequate intake (Food+Spp) of nutrients associated with bone health (such as calcium (53%), magnesium (47%), vitamin D (54%), and vitamin K (60%) (above AI)), which are significantly associated with short sleep duration. The relationships between sleep, nutrient intakes, and bone health warrant further investigation.

### 4.6. Other Micronutrients

Many micronutrients play a role in sleep and circadian regulation, including those that support melatonin synthesis (folate, vitamin B_6_, zinc) [39]. While the intake of these nutrients in U.S. adults (covariate adjusted UI or % below EAR) may not have been found to be significantly associated with short sleep in our analysis, it is plausible that threshold of intake is being met to fulfill the physiological needs. For instance, respondents may have individualized nutrient deficits that impair normal sleep regulation, but on average across the U.S. population intake levels may be enough to curb impediments to normal sleep and appear as a non-significant association in our analysis. This is an inherent limitation to the retrospective study design that cannot be addressed without a prospective intervention trial.

### 4.7. Study Limitations

There are limitations to this analysis. First, the retrospective study design only provides correlational data between nutrient intakes and short sleep, and odds ratios were not calculated. A nutrient intervention study using objective sleep measures is necessary to demonstrate causation. Next, sleep duration and diet records were self-reported and are limited by participant recall, under-reporting of dietary intakes, and perceptions of sleep on the day of questionnaire administration. In light of self-report limitations, the NHANES method for evaluating dietary intake patterns has been validated and continues to be used to track population-wide nutrient intakes [21]. The analysis also dichotomized sleep duration by short sleep duration (<7 h) and adequate sleep (>7 h), which grouped together short sleepers (<6 h) with individuals sleeping just a little below recommend levels (6–7 h) and normal sleepers (7–8 h) with long sleepers (>9 h). Inadequate nutrient intakes within these smaller sub-categories of sleep duration should be a focus of future research. In addition, the results are represented as weekday or workday sleep, which may have been different if the NHANES questionnaire had asked the question in the context of weekend sleep or an average across all days of the week.

Many other factors are also known to contribute to short sleep, including poor sleep hygiene, racial/ethnic disparities, and work shift policies. Many were addressed as covariates (age, gender, race/ethnicity, PIR level, education level, BMI, current smoking status, physical activity level, alcohol consumption, and energy intake) in our analysis on the basis of their significant association with sleep variables. Sleep duration is known to change throughout the lifespan and age-based recommendations for appropriate sleep recommendations have been made by the National Sleep Foundation [2], the American Academy of Sleep Medicine, and the Sleep Research Society [3]. Similarly, relationships between short sleep duration and race [53], sociodemographic status [54], BMI [55], cigarette smoking [56], alcohol consumption [57], and energy intake [15] have all been previously reported. One additional limitation is that we could not adjust the percentage below the EAR analyses for covariates like BMI and other physiological parameters. These types analyses are very difficult to conduct, as individual-based data are needed while the NCI methods are typically only used for population-based data. Many factors contribute to short sleep and holistic solutions to improving sleep may involve multiple approaches, one of which can include improving nutrient intake.

## 5. Conclusions

Using the NHANES 2005–2016, we have demonstrated a significant inverse association between the prevalence of short sleep and inadequate intake of several micronutrients, including calcium, magnesium, and vitamins A, C, D, and E. The DGA has identified these nutrients as under-consumed, and particularly vitamin D and calcium as nutrients of public concern. Although the demographics studied show more males than females with short sleep, the female participants showed a greater number of nutrients with inadequate intake associated with short sleep. Likewise, there were differences in inadequate nutrient intake across age groups and short sleep. Adults aged 51+ years showed a greater number of nutrients with intake below EAR associated with short sleep. Considering the high prevalence of sleep problems, including short sleep, and the high percentage of the population below the EAR for many of these nutrients, further work is needed to untangle the complex relationship and the potential impact of sleep and diet on optimal health. In addition, the association between short sleep and the usual intake of nutrients associated with bone health (including calcium, magnesium, and vitamins D and K) deserve further investigation. Because dietary recommendations are based on the prevention of deficiency, additional clinical studies are needed to determine if micronutrient status can be optimized for sleep health benefits.

## Figures and Tables

**Table 1:** Demographics of the study participants.

	All		Male		Female	
Characteristic	*n*	mean ± SE	*n*	mean ± SE	*n*	mean ± SE
Age (years)	26,287	47.12 ± 0.29 *	13,047	46.04 ± 0.32	13,240	48.17 ± 0.31
Female (%)	26,287	50.87 ± 0.36 *	13,047	100.00 ± 0.00	13,240	0.00 ± 0.00
Ethnicity						
Mexican American (%)	26,287	8.64 ± 0.85	13,047	9.32 ± 0.90	13,240	7.99 ± 0.82
Other Hispanic (%)	26,287	5.61 ± 0.54 *	13,047	5.48 ± 0.52	13,240	5.75 ± 0.57
Non-Hispanic White (%)	26,287	66.93 ± 1.58 *	13,047	67.07 ± 1.58 *	13,240	66.80 ± 1.65 *
Non-Hispanic Black (%)	26,287	11.41 ± 0.85 *	13,047	10.64 ± 0.77 *	13,240	12.16 ± 0.94 *
Other (%)	26,287	7.40 ± 0.47	13,047	7.50 ± 0.51	13,240	7.30 ± 0.49
Poverty-Income Ratio (PIR)						
≤1.35 (%)	24,028	24.28 ± 0.90 *	11,937	22.26 ± 0.94	12,091	26.24 ± 0.94 *
1.35> to <1.85 (%)	24,028	10.15 ± 0.36	11,937	9.85 ± 0.47	12,091	10.45 ± 0.35
≥1.85 (%)	24,028	65.56 ± 1.09 *	11,937	67.89 ± 1.18	12,091	63.32 ± 1.09 *
Education Level						
<High School Graduate (%)	26,186	16.73 ± 0.69	12,997	17.13 ± 0.78	13,189	16.34 ± 0.72 *
High School Graduate (%)	26,186	22.61 ± 0.60 *	12,997	23.42 ± 0.77 *	13,189	21.83 ± 0.65
>High School Graduate (%)	26,186	60.66 ± 1.05 *	12,997	59.45 ± 1.19 *	13,189	61.83 ± 1.07 *
Body Mass Index	25,929	28.89 ± 0.09 *	12,870	28.67 ± 0.10	13,059	29.10 ± 0.10 *
Smoking Current (%)	26,150	19.76 ± 0.60 *	12,975	23.01 ± 0.77 *	13,175	16.62 ± 0.60 *
Alcohol (drinks)	26,287	0.78 ± 0.02	13,047	1.10 ± 0.04	13,240	0.47 ± 0.02
Sleep Hours	26,211	7.08 ± 0.02 *	13,006	7.01 ± 0.02 *	13,205	7.14 ± 0.02 *
Short Sleep (%)	26,211	32.72 ± 0.53	13,006	34.42 ± 0.72	13,205	31.08 ± 0.66

* Significant difference (*p* < 0.001) between individuals with short sleep versus individuals without short sleep.

**Table 2:** Micronutrient usual intake (UI) (Food Only and Food + Spp) and inadequacy (% below estimated average requirement (EAR)) with and without short sleep duration in all adults.

	Usual Intake (Units)				EAR (% Below)			
	Food Only		Food + Supplement		Food Only		Food + Supplement	
	Short Sleep (Y)	Short Sleep (N)	Short Sleep (Y)	Short Sleep (N)	Short Sleep (Y)	Short Sleep (N)	Short Sleep (Y)	Short Sleep (N)
Calcium (mg)	953.08 ± 8.87	983.88 ± 7.41 *	1099.47 ± 11.16	1166.67 ± 10.74 *^#^	44.43 ± 0.96	42.10 ± 0.88	33.92 ± 0.89	29.59 ± 0.74 *
Copper (mg)	1.26 ± 0.01	1.32 ± 0.01 *	1.58 ± 0.02	1.68 ± 0.01 *	7.43 ± 0.47	5.23 ± 0.34 *	5.81 ± 0.39	3.82 ± 0.35 *
Folate, DFE (mcg)	528.30 ± 5.59	548.50 ± 4.05 *	726.19 ± 8.14	784.66 ± 9.44 *	14.1 ± 0.77	11.03 ± 0.58 *	10.19 ± 0.65	7.01 ± 0.52 *
Iron (mg)	14.80 ± 0.14	15.12 ± 0.07	17.88 ± 0.19	18.74 ± 0.19 *	5.93 ± 0.34	4.86 ± 0.20 *	4.65 ± 0.26	3.52 ± 0.20 *
Magnesium (mg)	296.41 ± 2.80	309.93 ± 2.07 *	321.31 ± 3.66	343.68 ± 4.65 *^#^	58.56 ± 1.12	50.91 ± 0.86 *	50.74 ± 1.08	42.61 ± 0.92 *
Niacin (mg)	26.19 ± 0.26	25.95 ± 0.15	34.36 ± 0.60	37.92 ± 0.84 *	1.56 ± 0.21	1.37 ± 0.15	1.06 ± 0.17	0.82 ± 0.11
Phosphorus (mg)	1374.93 ± 11.21	1400.31 ± 6.37	1384.72 ± 10.44	1414.23 ± 7.23	1.00 ± 0.15	0.66 ± 0.10	0.97 ± 0.13	0.62 ± 0.09
Riboflavin (mg)	2.14 ± 0.02	2.19 ± 0.01	4.40 ± 0.17	4.86 ± 0.24	3.41 ± 0.24	2.59 ± 0.18 *	2.77 ± 0.25	1.98 ± 0.16 *
Selenium (mcg)	113.60 ± 1.18	114.71 ± 0.63	127.09 ± 1.19	131.06 ± 1.03	0.65 ± 0.09	0.46 ± 0.06	0.50 ± 0.12	0.34 ± 0.07
Thiamin (mg)	1.60 ± 0.01	1.65 ± 0.01 *	5.00 ± 0.36	6.19 ± 0.53	7.72 ± 0.59	5.89 ± 0.42	5.78 ± 0.44	3.89 ± 0.32 *
Vitamin A (mcg)^1^	604.08 ± 9.89	655.41 ± 7.27 *	919.60 ± 14.34	1009.93 ± 19.09 *	50.87 ± 1.46	42.48 ± 0.99 *	39.00 ± 1.01	31.91 ± 0.98 *
Vitamin B12 (mcg)	5.19 ± 0.08	5.20 ± 0.05	58.32 ± 5.54	55.66 ± 3.63	4.36 ± 0.44	3.76 ± 0.31	3.02 ± 0.30	2.32 ± 0.17
Vitamin B6 (mg)	2.10 ± 0.03	2.13 ± 0.02	5.28 ± 0.25	5.46 ± 0.18	11.92 ± 0.84	10.53 ± 0.57	8.54 ± 0.65	6.65 ± 0.38
Vitamin C (mg)	77.73 ± 1.51	85.61 ± 1.21 *	155.93 ± 5.59	175.81 ± 4.28 *	50.68 ± 1.20	43.51 ± 1.00 *	37.56 ± 1.00	30.16 ± 0.78 *
Vitamin D (mcg)^2^	4.49 ± 0.07	4.81 ± 0.05 *	12.01 ± 0.39	14.93 ± 0.57 *^#^	95.70 ± 0.33	94.58 ± 0.35	68.39 ± 0.72	62.58 ± 0.69 *
Vitamin E (mg)^3^	8.25 ± 0.10	8.84 ± 0.09 *	25.23 ± 1.03	29.22 ± 1.38	86.04 ± 0.77	82.53 ± 0.81 *	62.35 ± 0.99	57.63 ± 0.84 *
Zinc (mg)	11.44 ± 0.11	11.69 ± 0.07	15.47 ± 0.20	16.20 ± 0.16 *	17.48 ± 0.94	14.42 ± 0.69 *	12.98 ± 0.71	10.10 ± 0.62 *
**Nutrients with AI, (% Above)**								
Potassium (mg)	2651.10 ± 23.93	2725.91 ± 14.89 *	2673.84 ± 23.09	2770.77 ± 15.67 *	2.28 ± 0.27	2.52 ± 0.21	2.56 ± 0.31	2.93 ± 0.26
Total choline (mg)	332.67 ± 2.74	337.07 ± 1.93	333.58 ± 2.55	338.07 ± 2.17	6.99 ± 0.56	7.79 ± 0.50	7.17 ± 0.76	8.01 ± 0.58
Vitamin K (mcg)	103.36 ± 2.03	114.42 ± 1.87 *	111.66 ± 3.02	119.93 ± 1.75	39.39 ± 1.42	47.79 ± 1.27 *	43.63 ± 1.20	51.23 ± 1.12 *

* Significantly different from Short Sleep (*p* < 0.01); ^#^ statistically significant (*p* < 0.01) after adjusting for covariates; ^1^ Vitamin A as mcg retinoic acid equivalents; ^2^ Vitamin D as mcg of Vitamin D_2_ + Vitamin D_3_; Vitamin E as mg of α-tocopherol. With Short Sleep, Yes (Y), Without Short Sleep, No (N).

**Table 3:** Micronutrient usual intake (UI) (Food Only and Food + Spp) and inadequacy (% below estimated average requirement (EAR)) with and without short sleep duration in females aged 19+ years.

	Usual Intake (Units)				EAR (% Below)			
	Food Only		Food + Supplement		Food Only		Food + Supplement	
	Short Sleep (Y)	Short Sleep (N)	Short Sleep (Y)	Short Sleep (N)	Short Sleep (Y)	Short Sleep (N)	Short Sleep (Y)	Short Sleep (N)
Calcium (mg)	817.64 ± 10.20	872.84 ± 8.62 *	1013.85 ± 15.08	1125.50 ± 12.33 *^#^	62.27 ± 1.42	56.32 ± 1.12 *	46.02 ± 1.37	37.14 ± 0.96 *
Copper (mg)	1.08 ± 0.01	1.17 ± 0.01 *	1.42 ± 0.03	1.54 ± 0.02 *	12.19 ± 0.93	7.84 ± 0.60 *	9.92 ± 0.77	6.01 ± 0.56 *
Folate, DFE (mcg)	441.35 ± 6.17	476.66 ± 4.85 *	672.25 ± 11.01	743.91 ± 12.36 *	22.02 ± 1.43	15.88 ± 0.96 *	15.72 ± 1.06	10.01 ± 0.69 *
Iron (mg)	12.31 ± 0.14	13.04 ± 0.10 *	16.66 ± 0.34	17.84 ± 0.26 *	11.84 ± 0.66	9.09 ± 0.32 *	9.33 ± 0.53	6.65 ± 0.36 *
Magnesium (mg)	252.32 ± 2.87	272.93 ± 2.18 *	279.89 ± 11.94	309.88 ± 6.08	59.58 ± 1.48	49.4 ± 1.08 *	51.2 ± 1.41	40.48 ± 1.03 *
Niacin (mg)	20.53 ± 0.23	21.13 ± 0.14	29.00 ± 0.88	31.94 ± 0.69 *	2.79 ± 0.42	2.22 ± 0.29	1.93 ± 0.34	1.36 ± 0.21
Phosphorus (mg)	1129.11 ± 9.95	1192.61 ± 8.95 *	1136.63 ± 9.44	1201.59 ± 8.85 *^#^	1.79 ± 0.31	1.05 ± 0.19	1.72 ± 0.28	1.00 ± 0.16
Riboflavin (mg)	1.76 ± 0.02	1.88 ± 0.02 *	4.32 ± 0.27	4.82 ± 0.35	4.08 ± 0.40	2.58 ± 0.28 *	3.22 ± 0.35	1.96 ± 0.22 *
Selenium (mcg)	91.24 ± 0.90	95.51 ± 0.56 *	102.84 ± 5.06	109.38 ± 3.79	1.24 ± 0.17	0.80 ± 0.11	1.01 ± 0.22	0.60 ± 0.14
Thiamin (mg)	1.32 ± 0.01	1.40 ± 0.01 *	5.08 ± 0.49	5.87 ± 0.53	12.09 ± 1.06	8.28 ± 0.72 *	8.97 ± 0.76	5.36 ± 0.56 *
Vitamin A (mcg) ^1^	544.15 ± 13.78	617.15 ± 8.16 *	893.63 ± 19.99	999.71 ± 22.81 *	49.3 ± 2.24	37.90 ± 1.18 *	36.84 ± 1.58	27.41 ± 1.09 *
Vitamin B12 (mcg)	4.07 ± 0.08	4.28 ± 0.05	70.47 ± 9.62	68.85 ± 4.18	7.65 ± 0.90	5.96 ± 0.62	5.27 ± 0.62	3.70 ± 0.37
Vitamin B6 (mg)	1.66 ± 0.02	1.76 ± 0.02 *	5.30 ± 0.41	5.41 ± 0.25	19.4 ± 1.28	15.35 ± 0.92	13.75 ± 1.13	9.86 ± 0.68 *
Vitamin C (mg)	70.27 ± 1.73	79.53 ± 1.77 *	157.58 ± 11.91	173.88 ± 7.1	48.81 ± 1.44	40.35 ± 1.26 *	34.85 ± 1.14	26.77 ± 0.94 *
Vitamin D (mcg) ^2^	3.82 ± 0.08	4.18 ± 0.06 *	13.47 ± 0.55	17.07 ± 0.95 *	98.52 ± 0.23	97.75 ± 0.25	66.18 ± 0.97	59.51 ± 0.87 *
Vitamin E (mg) ^3^	7.06 ± 0.10	7.98 ± 0.11 *	28.02 ± 1.58	30.27 ± 1.38	93.88 ± 0.67	89.17 ± 0.95 *	64.63 ± 1.1	59.60 ± 1.03 *
Zinc (mg)	9.19 ± 0.12	9.74 ± 0.08 *	13.52 ± 0.22	14.53 ± 0.15 *	19.43 ± 1.32	14.40 ± 0.83 *	14.27 ± 1.03	9.83 ± 0.62 *
**Nutrients with AI, (% Above)**								
Potassium (mg)	2241.94 ± 25.24	2384.35 ± 17.43 *	2256.44 ± 21.03	2415.34 ± 15.83 *	0.09 ± 0.03	0.20 ± 0.04	0.11 ± 0.03	0.22 ± 0.04
Total choline (mg)	263.95 ± 2.51	277.38 ± 2.26 *	264.39 ± 2.23	275.29 ± 2.37 *	2.69 ± 0.40	4.09 ± 0.52	2.66 ± 0.48	3.80 ± 0.61
Vitamin K (mcg)	99.90 ± 2.62	113.42 ± 2.35 *	110.95 ± 5.91	118.93 ± 2.05	47.25 ± 1.82	56.56 ± 1.50 *	51.20 ± 1.52	59.88 ± 1.25 *

* Significantly different from Short Sleep (*p* < 0.01); ^#^ statistically significant (*p* < 0.01) after adjusting for covariates; ^1^ Vitamin A as mcg retinoic acid equivalents; ^2^ Vitamin D as mcg of Vitamin D_2_ + Vitamin D_3_; ^3^ Vitamin E as mg of α-tocopherol. With Short Sleep, Yes (Y), Without Short Sleep, No (N).

**Table 4:** Micronutrient usual intake (UI) (Food Only and Food + Spp) and inadequacy (% below estimated average requirement (EAR)) with and without short sleep duration in males aged 19+ years.

	Usual Intake (Units)				EAR (% Below)			
	Food Only		Food + Supplement		Food Only		Food + Supplement	
	Short Sleep (Y)	Short Sleep (N)	Short Sleep (Y)	Short Sleep (N)	Short Sleep (Y)	Short Sleep (N)	Short Sleep (Y)	Short Sleep (N)
Calcium (mg)	1079.42 ± 10.93	1105.26 ± 9.76	1183.04 ± 13.18	1211.53 ± 13.02	27.64 ± 0.91	26.62 ± 0.91	22.07 ± 0.86	21.38 ± 0.82
Copper (mg)	1.42 ± 0.02	1.47 ± 0.01	1.72 ± 0.02	1.82 ± 0.02 *	2.99 ± 0.32	2.51 ± 0.26	2.02 ± 0.26	1.53 ± 0.22
Folate, DFE (mcg)	610.05 ± 7.75	626.44 ± 5.52	777.89 ± 11.55	828.41 ± 9.68 *	6.68 ± 0.55	5.79 ± 0.50	4.76 ± 0.52	3.85 ± 0.42
Iron (mg)	17.13 ± 0.18	17.38 ± 0.12	19.06 ± 0.30	19.73 ± 0.24	0.37 ± 0.08	0.27 ± 0.13	0.19 ± 0.05	0.19 ± 0.03
Magnesium (mg)	337.99 ± 3.73	350.13 ± 2.72 *	361.63 ± 8.42	380.22 ± 12.07	57.27 ± 1.31	52.61 ± 0.94 *	50.2 ± 1.32	44.89 ± 0.99 *
Niacin (mg)	31.52 ± 0.38	31.21 ± 0.25	39.53 ± 0.64	44.37 ± 1.51 *	0.34 ± 0.07	0.40 ± 0.08	0.22 ± 0.06	0.24 ± 0.05
Phosphorus (mg)	1604.52 ± 15.40	1627.01 ± 8.67	1626.43 ± 13.28	1644.8 ± 9.79	0.22 ± 0.05	0.20 ± 0.05	0.23 ± 0.05	0.22 ± 0.03
Riboflavin (mg)	2.50 ± 0.03	2.53 ± 0.02	4.46 ± 0.21	4.92 ± 0.24	2.80 ± 0.29	2.59 ± 0.23	2.22 ± 0.24	2.05 ± 0.21
Selenium (mcg)	134.59 ± 1.70	135.59 ± 1.03	150.71 ± 4.58	154.45 ± 5.23	0.08 ± 0.03	0.09 ± 0.02	0.05 ± 0.02	0.05 ± 0.02
Thiamin (mg)	1.87 ± 0.02	1.91 ± 0.01	4.93 ± 0.49	6.55 ± 0.94	3.76 ± 0.37	3.22 ± 0.34	2.81 ± 0.31	2.32 ± 0.29
Vitamin A (mcg) ^1^	659.82 ± 13.35	696.2 ± 11.04	944.66 ± 20.29	1021.05 ± 23.44	52.34 ± 1.43	47.62 ± 1.11 *	41.14 ± 1.28	36.79 ± 1.22
Vitamin B12 (mcg)	6.24 ± 0.14	6.20 ± 0.09	46.55 ± 5.16	41.44 ± 5.77	1.33 ± 0.30	1.38 ± 0.24	0.87 ± 0.24	0.83 ± 0.20
Vitamin B6 (mg)	2.51 ± 0.04	2.53 ± 0.02	5.27 ± 0.29	5.52 ± 0.21	5.04 ± 0.59	5.19 ± 0.55	3.50 ± 0.42	3.25 ± 0.31
Vitamin C (mg)	84.85 ± 2.52	92.29 ± 1.54	154.11 ± 11.53	177.78 ± 5.19	52.28 ± 1.81	46.86 ± 1.11	40.26 ± 1.73	33.89 ± 0.90 *
Vitamin D (mcg) ^2^	5.14 ± 0.10	5.51 ± 0.09 *	10.59 ± 0.51	12.63 ± 0.46 *	93.03 ± 0.56	91.12 ± 0.62	70.47 ± 0.85	65.92 ± 0.96 *
Vitamin E (mg) ^3^	9.35 ± 0.15	9.78 ± 0.11	22.54 ± 1.18	28.08 ± 2.11	78.75 ± 1.29	75.27 ± 1.01	60.10 ± 1.37	55.54 ± 0.98 *
Zinc (mg)	13.57 ± 0.16	13.81 ± 0.13	17.34 ± 0.35	17.99 ± 0.27	15.60 ± 1.08	14.40 ± 1.01	11.79 ± 0.77	10.44 ± 0.89
**Nutrients with AI, (% Above)**								
Potassium (mg)	3033.55 ± 34.93	3097.19 ± 19.03	3078.15 ± 36.37	3151.68 ± 22.65	4.35 ± 0.50	5.06 ± 0.43	4.95 ± 0.60	5.84 ± 0.55
Total choline (mg)	396.75 ± 4.20	401.98 ± 2.88	400.27 ± 3.73	405.75 ± 3.00	10.98 ± 0.94	11.86 ± 0.76	11.50 ± 1.18	12.54 ± 0.95
Vitamin K (mcg)	106.78 ± 2.17	115.66 ± 2.08 *	112.36 ± 2.29	120.76 ± 2.28 *	32.17 ± 1.50	38.27 ± 1.37 *	36.25 ± 1.65	41.77 ± 1.36 *

* Significantly different from Short Sleep (*p* < 0.01); ^1^ Vitamin A as mcg retinoic acid equivalents; ^2^ Vitamin D as mcg of Vitamin D_2_ + Vitamin D_3_; ^3^ Vitamin E as mg of α-tocopherol. With Short Sleep, Yes (Y), Without Short Sleep, No (N).

## Supplementary Materials

The following are available online at https://www.mdpi.com/2072-6643/11/10/2335/s1. Table S1. Micronutrient usual intake (UI) (Food Only and Food + Spp) and inadequacy (% below EAR) with and without short sleep in adults aged 19–50 years. Table S2. Micronutrient usual intake (UI) (Food Only and Food + Spp) and inadequacy (% below EAR) with and without short sleep in adults aged 51–99 years. Table S3. Micronutrient usual intake (UI) (Food Only and Food + Spp) and inadequacy (% below EAR) with and without short sleep in females aged 19–50 years. Table S4. Micronutrient usual intake (UI) (Food Only and Food + Spp) and inadequacy (% below EAR) with and without short sleep in females aged 51–99 years. Table S5. Micronutrient usual intake (UI) (Food Only and Food + Spp) and inadequacy (% below EAR) with and without short sleep in males aged 19–50 years. Table S6. Micronutrient usual intake (UI) (Food Only and Food + Spp) and inadequacy (% below EAR) with and without short sleep in males aged 51–99 years.
