# Supplementary material for: Micronutrient Inadequacy in Short Sleep: Analysis of the NHANES 2005–2016
Source: Nutrients. 2019 Oct 1;11(10):2335. doi: 10.3390/nu11102335 (PMC6835726; doi:10.3390/nu11102335)
Supplement: Supplementary file 1 [file nutrients-11-02335-s001.pdf]

Table S1. Micronutrient Usual Intake (UI) (Food Only and Food + Spp) and Inadequacy (% Below EAR) With and Without Short Sleep in Adults, 19-50 y

	Usual Intake				EAR (% Below)			
	Food Only		Food+Supplement		Food Only		Food+Supplement	
	Short Sleep (Y)	Short Sleep (N)	Short Sleep (Y)	Short Sleep (N)	Short Sleep (Y)	Short Sleep (N)	Short Sleep (Y)	Short Sleep (N)
Calcium (mg)	987.45 ± 10.47	1024.35 ± 9.11 *	1084.31 ± 13.04	1126.55 ± 11.56	35.36 ± 1.11	30.97 ± 1.02 *	29.29 ± 1.02	25.19 ± 0.87 *
Copper (mg)	1.27 ± 0.01	1.32 ± 0.01 *	1.53 ± 0.02	1.61 ± 0.02 *	7.41 ± 0.47	5.31 ± 0.4 *	6.23 ± 0.48	4.29 ± 0.42 *
Folate, DFE (mcg)	541.24 ± 6.78	562.74 ± 5.09	696.96 ± 8.90	734.91 ± 10.57 *	12.91 ± 0.80	9.86 ± 0.59 *	9.92 ± 0.74	7.12 ± 0.59 *
Iron (mg)	15.05 ± 0.17	15.40 ± 0.10	17.89 ± 0.24	18.51 ± 0.20	9.15 ± 0.50	8.19 ± 0.29	7.24 ± 0.39	5.98 ± 0.33
Magnesium (mg)	299.57 ± 3.29	313.17 ± 2.57 *	315.99 ± 4.59	334.15 ± 3.34 *#	57.23 ± 1.29	49.34 ± 1.08 *	51.79 ± 1.26	44.00 ± 1.08 *
Niacin (mg)	27.45 ± 0.31	27.41 ± 0.20	33.35 ± 0.75	33.66 ± 0.54	1.02 ± 0.17	0.82 ± 0.11	0.81 ± 0.16	0.59 ± 0.10
Phosphorus (mg)	1425.43 ± 13.10	1457.99 ± 8.07	1436.39 ± 14.63	1469.54 ± 8.57	0.72 ± 0.15	0.45 ± 0.08	0.71 ± 0.12	0.47 ± 0.08
Riboflavin (mg)	2.15 ± 0.02	2.20 ± 0.02	3.94 ± 0.20	4.21 ± 0.17	3.47 ± 0.33	2.66 ± 0.23	2.95 ± 0.28	2.25 ± 0.21
Selenium (mcg)	118.33 ± 1.41	120.32 ± 0.85	128.67 ± 1.36	132.37 ± 1.31	0.41 ± 0.08	0.26 ± 0.05	0.39 ± 0.09	0.24 ± 0.06
Thiamin (mg)	1.64 ± 0.02	1.69 ± 0.01	3.87 ± 0.24	4.35 ± 0.24	6.89 ± 0.59	5.01 ± 0.42 *	5.54 ± 0.47	3.77 ± 0.39 *
Vitamin A (mcg)[1]	580.03 ± 10.10	621.43 ± 8.81 *	812.28 ± 14.08	868.56 ± 17.47	54.95 ± 1.52	47.59 ± 1.26 *	44.40 ± 1.24	38.18 ± 1.17 *
Vitamin B12 (mcg)	5.31 ± 0.09	5.33 ± 0.06	33.13 ± 3.75	32.77 ± 4.41	4.19 ± 0.46	3.53 ± 0.30	3.09 ± 0.33	2.39 ± 0.21
Vitamin B6 (mg)	2.17 ± 0.03	2.20 ± 0.02	4.46 ± 0.21	4.63 ± 0.17	7.06 ± 0.69	5.60 ± 0.51	5.85 ± 0.63	4.32 ± 0.40
Vitamin C (mg)	75.19 ± 1.65	81.97 ± 1.46 *	132.65 ± 5.54	142.89 ± 5.33	53.26 ± 1.37	46.69 ± 1.26 *	42.50 ± 1.04	35.86 ± 1.07 *
Vitamin D (mcg)[2]	4.37 ± 0.08	4.62 ± 0.06	8.62 ± 0.26	10.89 ± 0.60 *#	96.08 ± 0.35	95.31 ± 0.35	76.04 ± 0.72	73.31 ± 0.76 *
Vitamin E (mg)[3]	8.27 ± 0.11	8.87 ± 0.11 *	19.65 ± 1.36	19.50 ± 0.79	85.72 ± 0.87	82.25 ± 0.87 *	66.87 ± 0.96	63.56 ± 0.99
Zinc (mg)	11.78 ± 0.13	12.07 ± 0.09	14.71 ± 0.18	15.09 ± 0.19	15.68 ± 0.97	12.56 ± 0.74	12.31 ± 0.86	9.62 ± 0.67
**Nutrients with AI, (% Above)**								
Potassium (mg)	2628.82 ± 26.8	2698.03 ± 19.03	2647.85 ± 25.92	2732.29 ± 18.97 *	2.26 ± 0.29	2.52 ± 0.24	2.51 ± 0.31	2.85 ± 0.31
Total choline (mg)	336.20 ± 3.41	341.05 ± 2.35	338.22 ± 3.61	342.09 ± 2.66	7.36 ± 0.63	8.23 ± 0.56	7.78 ± 0.95	8.49 ± 0.72
Vitamin K (mcg)	98.79 ± 2.13	108.45 ± 1.92 *	102.27 ± 2.01	111.76 ± 2.23 *#	35.81 ± 1.50	43.44 ± 1.33 *	38.62 ± 1.43	45.37 ± 1.43 *

*Significantly different from Short Sleep (p<0.01); #Usual intake statistically significant (p<0.01) after adjusting for covariates (age, gender, race/ethnicity, PIR level, education level, BMI, current smoking status, and physical activity level, IUI of alcohol, and IUI of energy); [1]Vitamin A as mcg retinoic acid equivalents;[2] Vitamin D as mcg of Vitamin D_2+Vitamin D_3; Vitamin E as mg of α-tocopherol

Table S2. Micronutrient Usual Intake (UI) (Food Only and Food + Spp) and Inadequacy (% Below EAR) With and Without Short Sleep in Adults 51-99 y

	Usual Intake (Units)				EAR (% Below)			
	Food Only		Food+Supplement		Food Only		Food+Supplement	
	Short Sleep (Y)	Short Sleep (N)	Short Sleep (Y)	Short Sleep (N)	Short Sleep (Y)	Short Sleep (N)	Short Sleep (Y)	Short Sleep (N)
Calcium (mg)	900.94 ± 8.91	933.59 ± 9.02	1123.54 ± 13.90	1216.05 ± 14.31 *#	58.15 ± 0.95	56.09 ± 1.04	41.08 ± 1.05	35.33 ± 0.96 *
Copper (mg)	1.24 ± 0.01	1.30 ± 0.01 *	1.64 ± 0.03	1.76 ± 0.03 *	7.54 ± 0.49	5.27 ± 0.38 *	5.33 ± 0.42	3.36 ± 0.32 *
Folate, DFE (mcg)	509.35 ± 5.78	530.89 ± 4.78 *	771.95 ± 13.70	848.74 ± 13.03 *	15.84 ± 0.92	12.48 ± 0.74	10.25 ± 0.70	7.01 ± 0.52 *
Iron (mg)	14.41 ± 0.14	14.78 ± 0.12	17.82 ± 0.30	19.04 ± 0.35 *	1.02 ± 0.17	0.68 ± 0.12	0.67 ± 0.13	0.40 ± 0.08
Magnesium (mg)	292.30 ± 2.90	305.73 ± 2.23 *	329.19 ± 4.37	356.10 ± 8.24 *	60.20 ± 1.24	52.89 ± 0.93 *	49.20 ± 1.32	40.88 ± 1.12 *
Niacin (mg)	24.31 ± 0.27	24.11 ± 0.17	35.93 ± 0.86	43.45 ± 1.84 *	2.29 ± 0.31	2.05 ± 0.23	1.49 ± 0.21	1.12 ± 0.13
Phosphorus (mg)	1301.83 ± 9.97	1329.15 ± 8.29	1307.42 ± 9.72	1344.63 ± 9.43 *	1.31 ± 0.19	0.90 ± 0.13	1.28 ± 0.18	0.83 ± 0.12
Riboflavin (mg)	2.14 ± 0.02	2.19 ± 0.02	5.09 ± 0.37	5.70 ± 0.45	3.26 ± 0.24	2.46 ± 0.19 *	2.37 ± 0.20	1.69 ± 0.15 *
Selenium (mcg)	106.56 ± 1.13	107.69 ± 0.80	124.50 ± 1.44	129.42 ± 1.19 *	1.00 ± 0.17	0.70 ± 0.11	0.73 ± 0.15	0.48 ± 0.10
Thiamin (mg)	1.55 ± 0.01	1.59 ± 0.01	6.74 ± 0.87	8.58 ± 1.17	9.32 ± 0.77	6.94 ± 0.52	6.25 ± 0.45	4.17 ± 0.34 *
Vitamin A (mcg)[1]	639.45 ± 11.69	696.90 ± 7.73 *	1088.77 ± 26.29	1195.31 ± 31.6 *	44.89 ± 1.68	36.32 ± 0.98 *	30.33 ± 1.18	23.76 ± 0.98 *
Vitamin B12 (mcg)	5.01 ± 0.09	5.04 ± 0.06	97.37 ± 11.61	85.29 ± 8.44	4.79 ± 0.57	4.09 ± 0.39	3.03 ± 0.34	2.20 ± 0.20
Vitamin B6 (mg)	2.00 ± 0.03	2.04 ± 0.02	6.57 ± 0.51	6.54 ± 0.30	19.44 ± 1.16	16.82 ± 0.81	12.52 ± 0.89	9.65 ± 0.50 *
Vitamin C (mg)	81.92 ± 1.90	90.32 ± 1.44 *	191.73 ± 11.26	218.23 ± 5.50	46.66 ± 1.39	39.33 ± 1.09 *	30.42 ± 1.44	22.95 ± 0.76 *
Vitamin D (mcg)[2]	4.70 ± 0.08	5.05 ± 0.07 *	17.28 ± 0.82	20.15 ± 0.88	95.02 ± 0.39	93.75 ± 0.46	56.43 ± 1.28	48.72 ± 1.11 *
Vitamin E (mg)[3]	8.18 ± 0.10	8.81 ± 0.10 *	33.90 ± 1.56	41.78 ± 2.89	86.68 ± 0.82	82.87 ± 0.93 *	55.30 ± 1.48	49.99 ± 1.22 *
Zinc (mg)	10.94 ± 0.13	11.22 ± 0.10	16.64 ± 0.44	17.63 ± 0.30	20.29 ± 1.17	16.78 ± 0.81	13.93 ± 0.82	10.60 ± 0.76 *
**Nutrients with AI, (% Above)**								
Potassium (mg)	2688.60 ± 24.71	2767.30 ± 17.19 *	2709.36 ± 22.61	2819.14 ± 18.63 *	2.40 ± 0.29	2.64 ± 0.23	2.49 ± 0.32	2.96 ± 0.27
Total choline (mg)	327.25 ± 3.06	332.07 ± 2.37	326.34 ± 2.65	332.80 ± 2.75	6.43 ± 0.52	7.30 ± 0.54	6.31 ± 0.63	7.42 ± 0.59
Vitamin K (mcg)	110.36 ± 2.38	121.72 ± 2.16 *	126.17 ± 7.48	130.17 ± 2.02	44.90 ± 1.68	53.19 ± 1.4 *	51.31 ± 1.15	58.65 ± 1.08 *

*Significantly different from Short Sleep (p<0.01); #Usual intake statistically significant (p<0.01) after adjusting for covariates (age, gender, race/ethnicity, PIR level, education level, BMI, current smoking status, and physical activity level, IUI of alcohol, and IUI of energy), [1]Vitamin A as mcg retinoic acid equivalents;[2] Vitamin D as mcg of Vitamin D_2+Vitamin D_3; Vitamin E as mg of α-tocopherol

Table S3. Micronutrient Usual Intake (UI) (Food Only and Food + Spp) and Inadequacy (% Below EAR) With and Without Short Sleep in Females 19-50 y

	Usual Intake (Units)				EAR (% Below)			
	Food Only		Food+Supplement		Food Only		Food+Supplement	
	Short Sleep (Y)	Short Sleep (N)	Short Sleep (Y)	Short Sleep (N)	Short Sleep (Y)	Short Sleep (N)	Short Sleep (Y)	Short Sleep (N)
Calcium (mg)	830.91 ± 11.54	888.76 ± 9.66 *	951.06 ± 13.95	1024.98 ± 12.45 *	50.17 ± 1.73	42.21 ± 1.37 *	41.04 ± 1.34	33.13 ± 1.3 *
Copper (mg)	1.07 ± 0.02	1.16 ± 0.01 *	1.35 ± 0.03	1.46 ± 0.02 *	12.81 ± 1.08	8.24 ± 0.71 *	11.15 ± 0.86	6.97 ± 0.71 *
Folate, DFE (mcg)	446.89 ± 7.32	483.23 ± 6.32 *	645.71 ± 15.36	685.37 ± 14.73	20.97 ± 1.59	15.03 ± 1.09 *	16.37 ± 1.22	10.6 ± 0.93 *
Iron (mg)	12.40 ± 0.16	13.15 ± 0.13 *	16.58 ± 0.40	17.61 ± 0.30	19.34 ± 0.98	16.15 ± 0.55 *	15.21 ± 0.79	11.77 ± 0.64 *
Magnesium (mg)	251.12 ± 3.41	271.64 ± 2.86 *	266.62 ± 14.83	291.39 ± 9.43	59.73 ± 1.76	49.17 ± 1.44 *	54.43 ± 1.61	43.88 ± 1.4 *
Niacin (mg)	21.10 ± 0.25	21.89 ± 0.16 *	27.78 ± 1.09	27.67 ± 0.52	2.07 ± 0.36	1.42 ± 0.23	1.59 ± 0.35	1.13 ± 0.22
Phosphorus (mg)	1148.36 ± 11.07	1216.33 ± 9.97 *	1151.09 ± 10.08	1217.87 ± 9.85 *	1.50 ± 0.30	0.82 ± 0.16	1.46 ± 0.25	0.82 ± 0.16
Riboflavin (mg)	1.74 ± 0.02	1.86 ± 0.02 *	3.98 ± 0.38	3.80 ± 0.19	4.39 ± 0.43	2.72 ± 0.31 *	3.79 ± 0.46	2.36 ± 0.31 *
Selenium (mcg)	93.58 ± 0.94	98.45 ± 0.70 *	101.48 ± 5.34	107.48 ± 4.24	0.88 ± 0.16	0.52 ± 0.09	0.73 ± 0.21	0.44 ± 0.12
Thiamin (mg)	1.34 ± 0.02	1.42 ± 0.01 *	4.13 ± 0.43	4.05 ± 0.32	11.18 ± 1.15	7.49 ± 0.78 *	9.08 ± 1.00	5.47 ± 0.72 *
Vitamin A (mcg)[1]	511.44 ± 14.53	576.57 ± 10.80 *	776.79 ± 22.45	832.50 ± 22.30	54.84 ± 2.47	43.69 ± 1.65 *	43.12 ± 1.93	34.27 ± 1.53 *
Vitamin B12 (mcg)	4.07 ± 0.08	4.29 ± 0.05	30.67 ± 4.26	46.43 ± 8.39	7.59 ± 0.83	5.99 ± 0.60	5.69 ± 0.67	4.12 ± 0.46
Vitamin B6 (mg)	1.67 ± 0.03	1.78 ± 0.02 *	4.38 ± 0.37	4.34 ± 0.34	13.25 ± 1.24	9.51 ± 0.82	10.88 ± 1.23	7.46 ± 0.72
Vitamin C (mg)	66.49 ± 1.72	74.73 ± 1.69 *	128.91 ± 19.06	133.09 ± 17.42	52.47 ± 1.75	44.37 ± 1.58 *	40.78 ± 1.51	32.87 ± 1.32 *
Vitamin D (mcg)[2]	3.63 ± 0.08	3.96 ± 0.07 *	8.94 ± 0.38	12.44 ± 1.31	98.89 ± 0.20	98.31 ± 0.21	75.04 ± 0.94	71.51 ± 1.10
Vitamin E (mg)[3]	6.95 ± 0.11	7.85 ± 0.13 *	22.44 ± 2.35	18.35 ± 0.99	94.41 ± 0.63	89.99 ± 0.92 *	70.44 ± 1.14	66.97 ± 1.27
Zinc (mg)	9.27 ± 0.11	9.87 ± 0.09 *	12.58 ± 0.28	13.01 ± 0.20	18.47 ± 1.39	13.33 ± 0.90 *	14.52 ± 1.15	10.20 ± 0.72 *
**Nutrients with AI, (% Above)**								
Potassium (mg)	2183.87 ± 26.64	2318.77 ± 20.82 *	2185.44 ± 22.27	2334.80 ± 19.43 *	0.06 ± 0.02	0.14 ± 0.04	0.08 ± 0.02	0.16 ± 0.04
Total choline (mg)	262.30 ± 2.55	275.46 ± 2.45 *	261.94 ± 2.49	271.93 ± 2.33 *	2.53 ± 0.37	3.85 ± 0.49	2.42 ± 0.47	3.39 ± 0.53
Vitamin K (mcg)	93.93 ± 2.76	106.16 ± 2.63 *	96.93 ± 2.23	109.18 ± 2.38 *	42.88 ± 2.01	51.97 ± 1.74 *	45.13 ± 1.68	53.72 ± 1.61 *

*Significantly different from Short Sleep ($p<0.01$); [1]Vitamin A as mcg retinoic acid equivalents;[2] Vitamin D as mcg of Vitamin D_2+Vitamin D_3; Vitamin E as mg of α-tocopherol

Table S4. Micronutrient Usual Intake (UI) (Food Only and Food + Spp) and Inadequacy (% Below EAR) With and Without Short Sleep in Females 51-99 y

	Usual Intake (Units)				EAR (Below %)			
	Food Only		Food+Supplement		Food Only		Food+Supplement	
	Short Sleep (Y)	Short Sleep (N)	Short Sleep (Y)	Short Sleep (N)	Short Sleep (Y)	Short Sleep (N)	Short Sleep (Y)	Short Sleep (N)
Calcium (mg)	801.05 ± 10.9	855.27 ± 9.92 *	1103.13 ± 22.75	1244.66 ± 18.5 *#	78.54 ± 1.35	72.43 ± 1.24 *	52.82 ± 1.78	41.81 ± 1.24 *
Copper (mg)	1.09 ± 0.02	1.18 ± 0.01 *	1.52 ± 0.03	1.65 ± 0.03 *	11.53 ± 1.01	7.37 ± 0.62 *	7.93 ± 0.79	4.84 ± 0.50 *
Folate, DFE (mcg)	435.15 ± 6.54	469.54 ± 5.85 *	711.35 ± 14.25	814.22 ± 16.14 *	23.27 ± 1.51	17.01 ± 1.16 *	14.78 ± 1.25	9.18 ± 0.74 *
Iron (mg)	12.22 ± 0.17	12.94 ± 0.15 *	16.71 ± 0.57	18.08 ± 0.46	1.53 ± 0.28	0.94 ± 0.17	1.04 ± 0.22	0.57 ± 0.14
Magnesium (mg)	254.20 ± 3.10	274.18 ± 2.54 *	297.57 ± 9.77	332.02 ± 7.26 *	59.68 ± 1.60	49.84 ± 1.29 *	47.16 ± 1.79	36.27 ± 1.36 *
Niacin (mg)	19.70 ± 0.25	20.29 ± 0.19	30.69 ± 1.33	37.00 ± 1.43 *	3.85 ± 0.56	3.10 ± 0.40	2.43 ± 0.37	1.75 ± 0.25
Phosphorus (mg)	1102.04 ± 11.91	1165.02 ± 10.53 *	1115.03 ± 11.88	1182.17 ± 11.45 *#	2.26 ± 0.36	1.38 ± 0.21	2.11 ± 0.35	1.20 ± 0.20
Riboflavin (mg)	1.79 ± 0.02	1.91 ± 0.02 *	4.81 ± 0.41	6.03 ± 0.75	3.66 ± 0.37	2.30 ± 0.29 *	2.55 ± 0.31	1.50 ± 0.18 *
Selenium (mcg)	88.10 ± 1.11	92.26 ± 0.76 *	104.39 ± 4.89	111.77 ± 3.45	1.76 ± 0.25	1.13 ± 0.15	1.30 ± 0.28	0.76 ± 0.17
Thiamin (mg)	1.30 ± 0.02	1.38 ± 0.01 *	6.41 ± 1.06	8.03 ± 1.01	13.30 ± 1.23	9.32 ± 0.87 *	8.85 ± 0.80	5.28 ± 0.50 *
Vitamin A (mcg)[1]	587.49 ± 15.12	664.14 ± 8.88 *	1060.16 ± 28.23	1200.82 ± 36.68 *	42.13 ± 2.41	31.01 ± 1.35 *	27.74 ± 1.59	19.54 ± 1.09 *
Vitamin B12 (mcg)	4.07 ± 0.09	4.28 ± 0.06	126.27 ± 21.09	95.53 ± 9.53	7.71 ± 1.07	6.10 ± 0.75	4.68 ± 0.56	3.22 ± 0.36
Vitamin B6 (mg)	1.65 ± 0.03	1.74 ± 0.02 *	6.60 ± 0.84	6.69 ± 0.47	28.11 ± 1.82	22.21 ± 1.21 *	17.62 ± 1.38	12.59 ± 0.87 *
Vitamin C (mg)	75.60 ± 1.39	85.06 ± 1.53 *	197.51 ± 17.45	222.48 ± 14.88	43.51 ± 1.35	35.57 ± 1.33 *	26.83 ± 1.24	19.50 ± 0.99 *
Vitamin D (mcg)[2]	4.07 ± 0.09	4.44 ± 0.08 *	19.84 ± 1.06	22.58 ± 1.18	98.03 ± 0.30	97.08 ± 0.34	53.80 ± 1.53	45.13 ± 1.33 *
Vitamin E (mg)[3]	7.23 ± 0.12	8.13 ± 0.13 *	35.87 ± 2.39	44.48 ± 2.71	93.08 ± 0.83	88.20 ± 1.15 *	56.50 ± 1.70	50.75 ± 1.55
Zinc (mg)	9.04 ± 0.12	9.60 ± 0.09 *	14.86 ± 0.34	16.32 ± 0.25 *	21.08 ± 1.56	15.61 ± 1.04 *	13.93 ± 1.16	9.25 ± 0.76 *
**Nutrients with AI, (% Above)**								
Potassium (mg)	2319.94 ± 28.65	2462.89 ± 20.43 *	2350.42 ± 28.40	2512.60 ± 23.87 *#	0.12 ± 0.04	0.24 ± 0.05	0.14 ± 0.04	0.31 ± 0.07
Total choline (mg)	266.71 ± 3.12	279.70 ± 2.79 *	267.52 ± 3.32	279.37 ± 3.34	3.00 ± 0.48	4.23 ± 0.63	2.83 ± 0.59	4.29 ± 0.67
Vitamin K (mcg)	107.18 ± 3.33	121.20 ± 2.79 *	131.08 ± 14.41	131.17 ± 2.96	52.78 ± 2.20	61.61 ± 1.71 *	59.82 ± 1.95	67.81 ± 1.46 *

*Significantly different from Short Sleep (p<0.01); #Usual intake statistically significant (p<0.01) after adjusting for covariates (age, gender, race/ethnicity, PIR level, education level, BMI, current smoking status, and physical activity level, IUI of alcohol, and IUI of energy); [1]Vitamin A as mcg retinoic acid equivalents;[2] Vitamin D as mcg of Vitamin D_2+Vitamin D_3; Vitamin E as mg of α-tocopherol

Table S5. Micronutrient Usual Intake (UI) (Food Only and Food + Spp) and Inadequacy (% Below EAR) With and Without Short Sleep in Males 19-50 y

	Usual Intake (Units)				EAR (% Below)			
	Food Only		Food+Supplement		Food Only		Food+Supplement	
	Short Sleep (Y)	Short Sleep (N)	Short Sleep (Y)	Short Sleep (N)	Short Sleep (Y)	Short Sleep (N)	Short Sleep (Y)	Short Sleep (N)
Calcium (mg)	1123.53 ± 12.38	1159.9 ± 11.97	1203.67 ± 15.79	1229.98 ± 16.62	22.28 ± 0.99	19.63 ± 1.01	18.65 ± 0.95	17.15 ± 0.98
Copper (mg)	1.44 ± 0.02	1.49 ± 0.01	1.69 ± 0.02	1.77 ± 0.03	2.83 ± 0.32	2.30 ± 0.27	1.93 ± 0.26	1.55 ± 0.25
Folate, DFE (mcg)	624.24 ± 8.95	642.95 ± 6.61	743.06 ± 12.08	785.1 ± 11.67	5.82 ± 0.52	4.84 ± 0.48	4.38 ± 0.52	3.58 ± 0.45
Iron (mg)	17.37 ± 0.22	17.67 ± 0.17	19.02 ± 0.34	19.40 ± 0.21	0.27 ± 0.11	0.19 ± 0.08	0.19 ± 0.02	0.19 ± 0.09
Magnesium (mg)	342.06 ± 4.16	355.20 ± 3.15	359.82 ± 8.25	377.21 ± 11.51	55.05 ± 1.46	49.48 ± 1.11 *	49.49 ± 1.51	44.00 ± 1.09 *
Niacin (mg)	32.95 ± 0.41	33.00 ± 0.33	38.31 ± 0.79	39.63 ± 0.94	0.14 ± 0.05	0.14 ± 0.05	0.11 ± 0.04	0.09 ± 0.03
Phosphorus (mg)	1666.05 ± 16.58	1700.80 ± 12.06	1691.36 ± 18.02	1721.17 ± 14.05	0.11 ± 0.03	0.09 ± 0.02	0.10 ± 0.03	0.09 ± 0.02
Riboflavin (mg)	2.51 ± 0.03	2.54 ± 0.02	3.91 ± 0.15	4.63 ± 0.25	2.75 ± 0.33	2.51 ± 0.31	2.29 ± 0.27	2.18 ± 0.26
Selenium (mcg)	139.80 ± 1.77	142.38 ± 1.33	153.02 ± 5.03	157.26 ± 5.92	0.03 ± 0.01	0.02 ± 0.01	0.02 ± 0.01	0.01 ± 0.01
Thiamin (mg)	1.91 ± 0.02	1.96 ± 0.02	3.65 ± 0.18	4.65 ± 0.31 *#	3.13 ± 0.39	2.65 ± 0.35	2.47 ± 0.37	2.08 ± 0.30
Vitamin A (mcg)[1]	638.26 ± 12.14	666.25 ± 11.55	845.41 ± 18.02	906.07 ± 23.47	55.29 ± 1.4	51.49 ± 1.33	45.52 ± 1.5	42.28 ± 1.35
Vitamin B12 (mcg)	6.39 ± 0.15	6.37 ± 0.10	35.33 ± 5.98	19.10 ± 1.57 *	1.16 ± 0.26	1.15 ± 0.22	0.72 ± 0.21	0.75 ± 0.21
Vitamin B6 (mg)	2.59 ± 0.04	2.63 ± 0.03	4.53 ± 0.38	4.93 ± 0.27	1.83 ± 0.35	1.67 ± 0.31	1.33 ± 0.26	1.14 ± 0.22
Vitamin C (mg)	82.66 ± 2.45	89.41 ± 1.78	135.71 ± 7.53	152.44 ± 6.80	53.92 ± 1.82	48.81 ± 1.34	44.25 ± 1.65	38.98 ± 1.29
Vitamin D (mcg)[2]	5.00 ± 0.12	5.29 ± 0.10	8.34 ± 0.32	9.34 ± 0.33	93.65 ± 0.58	92.30 ± 0.63	76.88 ± 1.13	75.07 ± 1.13
Vitamin E (mg)[3]	9.42 ± 0.16	9.88 ± 0.13	17.16 ± 1.12	20.66 ± 1.15	78.18 ± 1.43	74.49 ± 1.17	63.65 ± 1.35	60.16 ± 1.22
Zinc (mg)	13.95 ± 0.17	14.28 ± 0.14	16.62 ± 0.21	17.14 ± 0.26	13.27 ± 1.09	11.69 ± 1.05	10.43 ± 0.92	9.18 ± 0.88
**Nutrients with AI, (% Above)**								
Potassium (mg)	3013.28 ± 37.66	3077.34 ± 24.08	3059.10 ± 38.31	3129.07 ± 28.56	4.15 ± 0.53	4.92 ± 0.46	4.68 ± 0.58	5.54 ± 0.61
Total choline (mg)	400.14 ± 4.98	406.97 ± 3.83	406.53 ± 4.56	412.11 ± 4.61	11.46 ± 1.03	12.64 ± 0.91	12.53 ± 1.36	13.51 ± 1.21
Vitamin K (mcg)	102.95 ± 2.26	110.50 ± 2.05	107.02 ± 2.62	114.43 ± 2.41	29.55 ± 1.56	34.75 ± 1.38	32.62 ± 1.88	37.32 ± 1.49

*Significantly different from Short Sleep ($p<0.01$); #Usual intake statistically significant ($p<0.01$) after adjusting for covariates (age, gender, race/ethnicity, PIR level, education level, BMI, current smoking status, and physical activity level, IUI of alcohol, and IUI of energy); [1]Vitamin A as mcg retinoic acid equivalents;[2] Vitamin D as mcg of Vitamin D_2+Vitamin D_3; Vitamin E as mg of α-tocopherol

Table S6. Micronutrient Usual Intake (UI) (Food Only and Food + Spp) and Inadequacy (% Below EAR) With and Without Short Sleep in Males 51-99 y

	Usual Intake				EAR (% Below)			
	Food Only		Food+Supplement		Food Only		Food+Supplement	
	Short Sleep (Y)	Short Sleep (N)	Short Sleep (Y)	Short Sleep (N)	Short Sleep (Y)	Short Sleep (N)	Short Sleep (Y)	Short Sleep (N)
Calcium (mg)	1007.6 ± 14.04	1028.95 ± 13.43	1148.12 ± 18.13	1182.21 ± 17.36	36.41 ± 1.27	36.26 ± 1.35	28.04 ± 1.17	27.48 ± 1.18
Copper (mg)	1.40 ± 0.02	1.44 ± 0.01	1.78 ± 0.04	1.90 ± 0.03 *[#]	3.47 ± 0.42	2.85 ± 0.32	2.25 ± 0.30	1.61 ± 0.21
Folate, DFE (mcg)	587.69 ± 8.87	604.92 ± 7.06	839.50 ± 24.30	890.01 ± 15.26	8.08 ± 0.71	6.95 ± 0.63	5.37 ± 0.61	4.28 ± 0.48
Iron (mg)	16.69 ± 0.21	17.02 ± 0.16	19.00 ± 0.39	20.15 ± 0.44	0.53 ± 0.12	0.37 ± 0.15	0.27 ± 0.11	0.19 ± 0.06
Magnesium (mg)	332.66 ± 4.25	343.83 ± 3.23	363.09 ± 9.49	384.90 ± 13.69	60.67 ± 1.53	56.66 ± 1.15	51.94 ± 1.67	46.25 ± 1.29 *
Niacin (mg)	29.11 ± 0.40	28.77 ± 0.25	41.62 ± 1.34	51.12 ± 3.41 *[#]	0.66 ± 0.12	0.75 ± 0.15	0.42 ± 0.10	0.43 ± 0.09
Phosphorus (mg)	1508.09 ± 15.59	1526.72 ± 10.16	1518.93 ± 14.36	1537.84 ± 13.81	0.37 ± 0.09	0.38 ± 0.08	0.45 ± 0.06	0.40 ± 0.07
Riboflavin (mg)	2.50 ± 0.03	2.53 ± 0.02	5.41 ± 0.58	5.31 ± 0.37	2.87 ± 0.28	2.64 ± 0.21	2.09 ± 0.26	1.94 ± 0.23
Selenium (mcg)	125.89 ± 1.73	126.42 ± 1.27	146.53 ± 4.08	150.49 ± 4.67	0.18 ± 0.05	0.16 ± 0.05	0.10 ± 0.04	0.08 ± 0.05
Thiamin (mg)	1.81 ± 0.02	1.84 ± 0.02	7.11 ± 1.37	9.24 ± 2.23	4.87 ± 0.52	4.19 ± 0.40	3.41 ± 0.38	2.87 ± 0.30
Vitamin A (mcg)	693.99 ± 17.76	736.4 ± 14.23	1119.16 ± 41.29	1188.81 ± 37.75	48.04 ± 1.93	42.70 ± 1.31	33.19 ± 1.65	28.84 ± 1.31
Vitamin B12 (mcg)	6.00 ± 0.16	5.97 ± 0.09	65.77 ± 8.79	73.11 ± 13.66	1.66 ± 0.36	1.70 ± 0.31	1.07 ± 0.27	0.97 ± 0.21
Vitamin B6 (mg)	2.38 ± 0.04	2.40 ± 0.02	6.54 ± 0.69	6.36 ± 0.43	10.71 ± 1.03	10.08 ± 0.92	7.09 ± 0.82	6.12 ± 0.52
Vitamin C (mg)	88.60 ± 3.27	96.64 ± 2.16	185.21 ± 9.16	213.03 ± 8.06	49.70 ± 2.24	43.78 ± 1.53	34.10 ± 2.41	27.17 ± 0.94 *
Vitamin D (mcg)	5.37 ± 0.12	5.78 ± 0.11	14.48 ± 1.29	17.27 ± 0.96	91.85 ± 0.68	89.68 ± 0.80	59.32 ± 1.59	52.94 ± 1.35
Vitamin E (mg)	9.17 ± 0.16	9.63 ± 0.13	31.78 ± 2.27	38.57 ± 4.94	80.00 ± 1.43	76.47 ± 1.22	53.77 ± 2.19	49.00 ± 1.44
Zinc (mg)	12.93 ± 0.19	13.18 ± 0.16	18.58 ± 0.89	19.18 ± 0.50	19.63 ± 1.35	18.08 ± 1.20	14.15 ± 0.98	12.18 ± 1.12
**Nutrients with AI, (% Above)**								
Potassium (mg)	3071.22 ± 36.65	3134.1 ± 22.86	3100.19 ± 41.11	3182.27 ± 25.35	4.80 ± 0.58	5.53 ± 0.49	5.05 ± 0.67	6.12 ± 0.61
Total choline (mg)	390.24 ± 4.57	395.02 ± 3.17	390.45 ± 4.63	396.51 ± 3.38	9.98 ± 0.90	10.79 ± 0.78	10.00 ± 0.95	11.16 ± 0.80
Vitamin K (mcg)	113.22 ± 2.78	122.03 ± 2.66	121.08 ± 2.37	129.76 ± 2.89	36.44 ± 1.95	42.65 ± 1.78	42.06 ± 1.68	48.03 ± 1.66

*Significantly different from Short Sleep ($p<0.01$); [#]Usual intake statistically significant ($p<0.01$) after adjusting for covariates (age, gender, race/ethnicity, PIR level, education level, BMI, current smoking status, and physical activity level, IUI of alcohol, and IUI of energy); [1]Vitamin A as mcg retinoic acid equivalents;[2] Vitamin D as mcg of Vitamin D_2+Vitamin D_3; Vitamin E as mg of α-tocopherol
